# Nursing Home Social Work With Residents With Severe Mental Illness, Thoughts of Suicide, or Dementia

**DOI:** 10.1093/geroni/igab046.444

**Published:** 2021-12-17

**Authors:** Mercedes Bern-Klug, Amy Restorick Roberts

## Abstract

Many of the close to 3 million persons who receive care in a U.S. nursing home in any given year face mental-health-related challenges that range from minor to severe. One of the core professionals involved with care planning for the psychosocial needs of nursing home residents with mental health concerns is the social worker. Reporting data from a 2019 nationally representative survey of nursing home social services directors, this session provides information about the training needs of nursing home social workers in terms of their work with residents diagnosed with a severe mental illness such as schizophrenia or severe depression, residents who are suicidal, and residents with dementia.

